# The genetic basis of structural colour variation in mimetic *Heliconius* butterflies

**DOI:** 10.1098/rstb.2020.0505

**Published:** 2022-07-18

**Authors:** Melanie N. Brien, Juan Enciso-Romero, Victoria J. Lloyd, Emma V. Curran, Andrew J. Parnell, Carlos Morochz, Patricio A. Salazar, Pasi Rastas, Thomas Zinn, Nicola J. Nadeau

**Affiliations:** ^1^ Ecology and Evolutionary Biology, School of Biosciences, The University of Sheffield, Alfred Denny Building, Western Bank, Sheffield S10 2TN, UK; ^2^ Biology Program, Faculty of Natural Sciences, Universidad del Rosario, Bogotá, Colombia; ^3^ Department of Physics and Astronomy, The University of Sheffield, Hicks Building, Hounsfield Road, Sheffield S3 7RH, UK; ^4^ Biology and Research Department, Mashpi Lodge, Ecuador; ^5^ Institute of Biotechnology, 00014 University of Helsinki, Finland; ^6^ ESRF - The European Synchrotron, 38043 Grenoble Cedex 9, France

**Keywords:** structural colour, iridescence, gene expression, *Heliconius*, QTL, convergence

## Abstract

Structural colours, produced by the reflection of light from ultrastructures, have evolved multiple times in butterflies. Unlike pigmentary colours and patterns, little is known about the genetic basis of these colours. Reflective structures on wing-scale ridges are responsible for iridescent structural colour in many butterflies, including the Müllerian mimics *Heliconius erato* and *Heliconius melpomene.* Here, we quantify aspects of scale ultrastructure variation and colour in crosses between iridescent and non-iridescent subspecies of both of these species and perform quantitative trait locus (QTL) mapping. We show that iridescent structural colour has a complex genetic basis in both species, with offspring from crosses having a wide variation in blue colour (both hue and brightness) and scale structure measurements. We detect two different genomic regions in each species that explain modest amounts of this variation, with a sex-linked QTL in *H. erato* but not *H. melpomene.* We also find differences between species in the relationships between structure and colour, overall suggesting that these species have followed different evolutionary trajectories in their evolution of structural colour. We then identify genes within the QTL intervals that are differentially expressed between subspecies and/or wing regions, revealing likely candidates for genes controlling structural colour formation.

This article is part of the theme issue ‘Genetic basis of adaptation and speciation: from loci to causative mutations’.

## 1. Introduction

Structural colours are some of the most vivid and striking colours found in nature. They are formed from the reflection and refraction of light from physical ultrastructures and examples of these can be found in nearly all groups of organisms. The structural colours of butterflies and moths are among the best described and play diverse roles, including initiation of courtship and mating behaviour [1,2], sex and species discrimination [3], long-distance mate recognition [4] signalling of quality and adult condition [5], and possibly predator avoidance [6,7].

**Figure 1.**
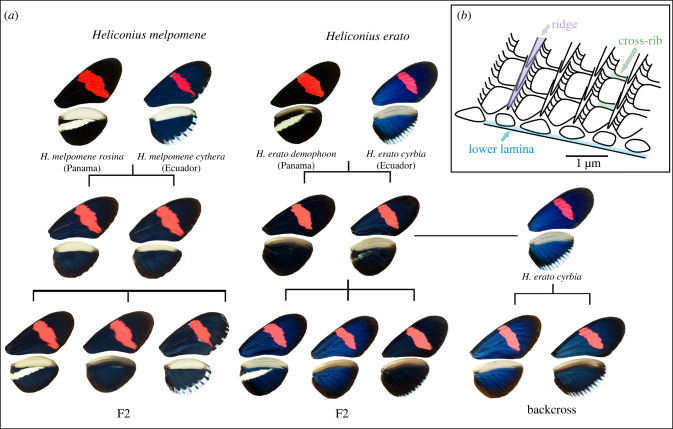
(*a*) Crosses between iridescent and non-iridescent morphs of *Heliconius melpomene* and *Heliconius erato*. For *H. melpomene*, we used F2 crosses, plus one cross thought to be F1 × F2 (not shown). For *H. erato*, we used F2 crosses and a backcross to the iridescent subspecies. (*b*) Schematic of part of a scale showing the lower lamina (blue) and upper longitudinal ridges (purple) connected by cross-ribs (green).

Butterflies and moths have evolved several mechanisms of structural colour production by modifying different components of wing-scale morphology [8,9]. Scales typically consist of a flat lower lamina connected to an upper lamina by pillar-like trabeculae, with a small space separating the upper and lower laminae (figure 1). The lower lamina can act as a thin film reflector that produces hues ranging from violet to green depending on its thickness [10–12]. The upper-scale surface has a more complex structure; it consists of a parallel array of ridges connected by cross-ribs, and modifications to these can yield diverse optical effects. For example, a lamellar structure in the ridges forms multi-layer reflectors that produce the iridescent (angle-dependent) blue in *Morpho* butterflies [13] and UV reflectance in *Colias eurytheme* [14,15]. The variations in hue and brightness of colour produced in the intricate structures of the upper-scale surface depend on an interplay between the number of lamellae, the thickness of each layer and the spacing between the ridges [16].

Recent studies have begun to uncover the genetic and developmental basis of structural colours in some species [9], revealing a common pattern in *Bicyclus anynana* and *Junonia coenia*; artificial selection for colourful phenotypes quickly resulted in changes in lower lamina thickness, and consequently hue, in a relatively small number of generations [10,12]. Knock-outs of known colour pattern genes [17], and genes involved in pigment synthesis pathways [18,19], have shown that modification of these can result in altered scale ultrastructure and, moreover, have brought about unexpected instances of the structural colour [18]. Interestingly, there are butterflies (*Junonia coena*) for which the gene *optix,* a known major colour pattern gene [20]*,* can jointly control pigment-based coloration and thickness of the lower lamina, producing blue structural colour [12]. Knock-outs of *optix* do not have an effect on structural colour in *Heliconius* [18]. Furthermore, the microevolutionary changes required for structural colour evolution are largely unknown.

Wing colour patterns have been widely studied in the *Heliconius* butterflies, a group of butterflies with a diverse set of aposematic colour patterns. These patterns show examples of both convergent evolution between distantly related species and divergent evolution within species. Some species form mimicry rings, in which wing patterning is under strong positive frequency-dependent selection due to predation [21]. Pigment colour patterns are largely determined by a small number of genes that are homologous across species. Extensive research has uncovered a toolkit of five loci that control much of the colour pattern variation in *Heliconius* species and some other Lepidoptera [22]. *Heliconius* also display structural colour, and in comparison to the well-studied pigmentary colours, very little is known about the development and genetic basis of these. While overall scale morphology is similar between iridescent and non-iridescent scales in *Heliconius*, those with blue structural colour have overlapping ridge lamellae that act as multi-layer reflectors (as in *Morpho*), along with a greater density of ridges on the scale (narrower ridge spacing) [16,23].

Structural colour has evolved multiple times within the *Heliconius* genus [16]. In some species, all subspecies have iridescent colour, while others exhibit interspecific variation in iridescence. *Heliconius erato* and *Heliconius melpomene* are two co-mimicking species that diverged around 10–13 Mya [24], with each evolving around 25 different colour pattern morphs [25]. Most of the different colour patterns are produced by pigment colours, but subspecies found west of the Andes in Ecuador and Colombia also have an iridescent blue structural colour. *H. erato cyrbia* and *H. melpomene cythera* found in Western Ecuador have the brightest iridescence, while subspecies *H. erato demophoon* and *H. melpomene rosina,* found to the north in Panama, are matt black in the homologous wing regions (figure 1). A hybrid zone forms between the iridescent and non-iridescent groups where they meet near the border between Panama and Colombia, and here, populations with intermediate levels of iridescence can be found [26]. Continuous variation in iridescent colour is observed in the centre of the hybrid zone and in experimental crosses [23], suggesting that this trait is controlled by multiple genes. The evolution of pigmentation and simple colour pattern traits has frequently been shown to involve the reuse of a small number of genes across animal species [22,27,28]. However, we may expect the genetic basis of a quantitative trait controlled by multiple genes, such as iridescence in these species, to be less predictable [29]. In addition, iridescence in *H. e. cyrbia* is much brighter than in *H. m. cythera* [16], suggesting some differences in scale structure and presumably genetic control of this structure formation process.

Here, we use crosses between subspecies of iridescent and non-iridescent *Heliconius* to determine the genetics of both colour and scale ultrastructure traits for the first time. We measure the intensity of blue colour and overall luminance (brightness) to assess variation in colour. We complement our estimates of colour variation with high-throughput measurements of ridge spacing and cross-rib spacing using ultra-small-angle X-ray scattering (USAXS). Using a quantitative trait locus (QTL) mapping approach, we can identify the location and effect sizes of loci in the genome that are controlling variation in iridescent colour. We then use RNA sequencing data from the same subspecies of each species to identify genes that are differentially expressed (DE), both between subspecies and between wing regions that differ in scale type. Comparison of the genetic basis of these traits between *H. melpomene* and *H. erato*, two distantly related mimetic species, allows us to ask whether, like pigment colour patterns, variation in iridescent colour and scale structure is also an example of gene reuse.

## 2. Methods

### (a) Experimental crosses

Experimental crosses were performed using geographical morphs of both *Heliconius erato* and *Heliconius melpomene*. In both species, morphs from Panama (*H. e. demophoon* and *H. m. rosina*) were crossed with morphs from Western Ecuador (*H. e. cyrbia* and *H. m. cythera*), then the F1 generations crossed with each other to produce an F2. For *H. erato*, we also analysed a backcross between the F1 and *H. e. cyrbia* (figure 1). Due to a mix-up in the insectary, one of our largest *H. melpomene* broods, named ‘EC70’, was obtained from a cross between an F1 father and a mother of unknown parentage, likely an F2 individual. Further details of the crosses are in the electronic supplementary material, table S1. A total of 155 *H. erato* individuals from five broods were used to generate linkage maps and perform QTL mapping (3 *demophoon* and 3 *cyrbia* grandparents, 11 F1 parents and 40 backcross and 99 F2 offspring). For *H. melpomene*, data from four broods made up of 228 individuals were used (1 *rosina* and 2 *cythera* grandparents, 6 parents and 219 offspring, electronic supplementary material, table S1). Some of these crosses have previously been used for an analysis of quantitative pattern variation [30]. Details of sequencing and linkage map construction are given in Bainbridge *et al.* [30] and in the electronic supplementary material.

### (b) Phenotypic measurements

In the offspring of these crosses, we measured four phenotypes—blue colour (BR), luminance, ridge spacing and cross-rib spacing. Wings were photographed under standard lighting conditions (full details in [23]). A colour checker in each photograph was used to standardize the photographs using the levels tool in Adobe Photoshop (CS3). RGB values (red, green and blue) were extracted from two blue/black areas of each wing (proximal areas of both the forewing and hind-wing, electronic supplementary material, figure S1) and averaged. Blue-red (BR) values were used as a measure of blue iridescent colour. These were calculated as (B − R)/(B + R), where 1 is completely blue and −1 is completely red. Luminance was measured as overall brightness and was calculated as R + G + B, with each colour having a maximum value of 255.

Scale structure measurements were extracted from USAXS data, from a single family of each species (*n* = 56 *H. erato* F2 and *n* = 73 *H. melpomene* (mother of unknown ancestry)). We measured between 33 and 113 points per individual along a linear proximodistal path across the proximal part of the forewing, which has the most vivid iridescence in the blue subspecies (electronic supplementary material, figure S1). The raw images were corrected for dark current and spatial distortion. SEM data from a subset of individuals were used to interpret the scattering patterns and develop robust methods for extracting mean ridge and cross-rib spacing values for the dorsal wing scales of all individuals (see electronic supplementary material for details).

### (c) Quantitative trait locus mapping

The R package R/qtl was used for the QTL analysis [31]. For *H. erato*, initially the F2 crosses were analysed together and the backcross analysed separately. Genotype probabilities were calculated for these two groups using *calc.genoprob* in R/QTL. We ran standard interval mapping to estimate LOD (logarithm of the odds) scores using the *scanone* function with the Haley-Knott regression method. In the F2 analysis, sex and family were included as additive covariates, and family was included as an interactive covariate, to allow multiple families to be analysed together. Sex was included as a covariate in the backcross analysis to account for any sexual dimorphism. To determine the significance level for the QTL, we ran 1000 permutations, with *perm.Xsp* *=* *T* to get a separate threshold for the Z chromosome. A single F2 family (*n* = 56) was used to analyse scale structure variation (ridge spacing and cross-rib spacing) using the same method, albeit that a higher number of permutations was used for determining the significance level of the QTL (4000). For analyses of BR colour and luminance, LOD scores for the F2 crosses and the backcross were added together, to allow analysis of all individuals together to increase power, and the significance level recalculated in R/qtl.

Confidence intervals for the positions of QTL were determined with the *bayesint* function and we used a *fitqtl* model to calculate the phenotypic variance that each QTL explained. Genome scan plots and genotype plots were made with R/qtl2 [32]. Genetic distances in the QTL results are based on the observed recombination rate and expressed in centimorgans (cM), which is the distance between two markers that recombine once per generation. These were related to physical distances based on the marker positions in the assembled reference genome of each species. Where we discuss individual markers, these are the markers with the highest LOD scores in each QTL.

The same method was used to run genome scans for BR colour and luminance in *H. melpomene*. Since the parentage of the mother of the EC70 brood is unknown, the maternal alleles in the offspring could not be assigned as being from either a *cythera* or a *rosina* grandparent. Therefore, in this family, only paternal alleles were taken into account (and all maternal alleles were assigned to a *rosina* grandparent), and the cross was treated as if a backcross. LOD scores of the three F2 families were added to the LOD score from the EC70 family, as in *H. erato*, and the significance level recalculated. Again, a single family was used for analysis of scale structures (EC70, *n* = 73).

### (d) Gene expression analysis

RNA sequence data were generated from 32 *H. erato* pupal wing samples (16 *H. e. demophoon and* 16 *H. e. cyrbia*) and *H. melpomene* pupal wing samples (16 *H. m. rosina* and 16 *H. m. cythera*), with individuals sampled from the same captive populations as those used for the crosses. Each of these samples contained two wing regions (the anterior hind-wing or ‘androconial’ region, which has a different scale type, was dissected from the rest of the wing and sampled separately; electronic supplementary material, figure S1), and two developmental stages, 50% total pupation time (5 days post-pupation) and 70% total pupation time (7 days post-pupation). Overall this gave four biological replicates for each tissue type/developmental stage/subspecies combination (electronic supplementary material, table S2).

Quality-trimmed reads were aligned to the respective *Heliconius* reference genomes using HISAT2 (v. 2.1.0) (see https://doi.org/10.1038/s41587-019-0201-4). Clustering of samples by multi-dimensional scaling (MDS) on expression levels revealed that one of the *H. m. rosina* individuals had been incorrectly labelled (which was also confirmed by analysis of nucleotide variants) and this was removed from subsequent analyses. Each species was analysed separately to identify genes that were DE between subspecies and between the wing regions for the iridescent blue subspecies (electronic supplementary material, figure S1), using the quasi-likelihood F-test in R/Bioconductor package EdgeR (v.3.28.1) (see https://doi.org/10.1093/bioinformatics/btp616). For the wing region comparison, we used a general linear model approach, with the two wing regions nested within ‘individual ID’ for each individual. We then determined if any significantly DE genes (between subspecies or wing region) were within the mapped QTL intervals. We further determined if any genes were DE in parallel between species. Details of further analyses of these data including gene set enrichment analysis are given in the electronic supplementary material.

## 3. Results

### (a) Quantitative trait locus mapping in *Heliconius erato*

**Figure 2.**
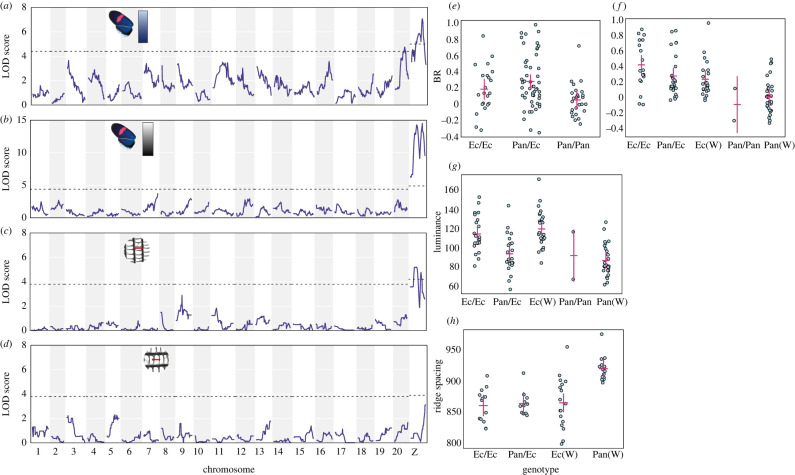
*H. erato* QTL analysis of all families for BR colour (*a*) and luminance (*b*), and for a single family for ridge spacing (*c*) and cross-rib spacing (*d*). Dotted lines show *p* = 0.05 significance level; (*e*–*h*) show the phenotypes of F2 individuals grouped by genotype at the most significant marker within each QTL ((*e*) top BR marker on chromosome 20; (*f*) top BR marker on Z; (*g*) top luminance marker on Z; (*h*) top ridge spacing marker on Z). ‘Pan’ denotes alleles from the Panama subspecies *demophoon*, and ‘Ec’ the Ecuador subspecies *cyrbia*. Only two individuals have homozygous Panama-type *demophoon* genotypes at the Z chromosome marker due to the small number of individuals with a *demophoon* maternal grandfather (electronic supplementary material, table S1). Points are individuals, red crosses show confidence intervals. Marker positions are shown in table 1.

We found significant correlations between scale structure and colour measurements: ridge spacing is negatively correlated with both luminance and BR values (electronic supplementary material, figure S2). Cross-rib spacing is positively correlated with ridge spacing and also negatively correlates with BR values (electronic supplementary material, text). Significant QTL were found for three phenotypes in *H. erato*—BR colour, luminance and ridge spacing (figure 2, table 1; electronic supplementary material, table S3). When analysing the colour measurements, F2 and backcross genome scans were combined, and for BR values, these showed two significant QTL on chromosomes 20 and the Z sex chromosome. These QTL were also found when analysing the F2 broods separately from the backcross brood (electronic supplementary material, figure S3). At both markers, individuals with Panama-type genotypes (Pan/Pan and Pan(W)) had lower BR values than Ecuador-type and heterozygous genotypes, following the expected trend (figure 2). The QTL on the Z chromosome explained the largest proportion of the phenotypic variation in BR colour in both the F2 crosses (19.5%) and the backcross (24.6%), and the chromosome 20 QTL explained a further 12.3% in the F2 crosses.

**Table 1.** Significant QTL were found for three phenotypes in *H. erato* and *H. melpomene*.

phenotype	marker	chromosome	position (cM)	LOD	*p*
*Heliconius erato*					
BR colour (all families)	Herato2101_12449252	Z	38.0	7.07	0.001
	Herato2001_12633065	20	32.9	4.75	0.022
luminance (all families)	Herato2101_12449398	Z	41.6	14.50	<0.001
ridge spacing (single family)	Herato2101_7491127	Z	23.0	5.21	0.013
*Heliconius melpomene*					
BR (all families)	Hmel203003o_2119654	3	15.22	7.26	0.001
luminance (all families)	Hmel203003o_2635435	3	17.97	13.61	<0.001
ridge spacing (EC70)	Hmel207001o_11550301	7	53.61	5.71	<0.001

Luminance (overall brightness of the wing region) was highly associated with the Z chromosome (figure 2*b*). The significant marker did not map exactly to the same position as for the BR values but was apart by only 3.6 cM, and confidence intervals for each overlap. Individuals with Ecuador-type alleles had higher luminance values than those with Panama-type alleles, showing the same trend as the BR values (figure 2*g*). This QTL explained 40.2% of the variance in luminance values in the F2 crosses and 24.2% in the backcross. This was the only significant QTL for luminance, with nothing appearing on chromosome 20.

A single QTL on the Z chromosome was also significant for ridge spacing (figure 2*c*). This marker was at a different position from the markers for BR and luminance, but mapped to the same marker as luminance when using the same individuals (electronic supplementary material, figure S3). All genotypes with one or two Ecuador-type alleles had similar ridge spacing, but those with a hemizygous Panama-type genotype (‘Pan(W)’ in figure 2*h*) had significantly wider ridge spacing. This QTL explained 34.8% of variance in ridge spacing in this family. No significant QTL were found for cross-rib spacing, although the highest LOD score was seen on the Z chromosome (figure 2*d*).

### (b) Quantitative trait locus mapping in *Heliconius melpomene*

**Figure 3.**
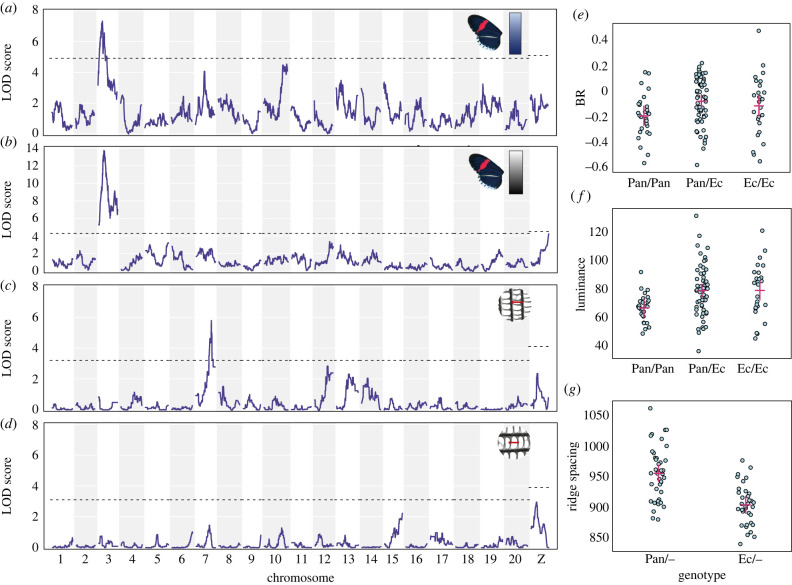
*Heliconius melpomene* QTL analysis of all families for BR colour (*a*) and luminance (*b*), and for a single family (EC70) for ridge spacing (*c*) and cross-rib spacing (*d*); (*e*–*g*) show the phenotypes of individuals grouped by genotype at the most significant marker within each QTL ((*e*) BR colour of F2 individuals by chromosome 3 marker genotype; (*f*) luminance of F2 individuals by chromosome 3 marker genotype; (*g*) Ridge spacing of EC70 brood individuals by chromosome 7 marker genotype). ‘Pan’ denotes alleles from the Panama subspecies *rosina*, and ‘Ec’ the Ecuador subspecies *cythera*. Points are individuals, red crosses show confidence intervals. Marker positions are shown in table 1.

In contrast with *H. erato*, scale structure measurements in *H. melpomene* did not correlate with either of the colour measurements (supplementary material, figure S2, text). A single significant QTL for BR colour was found on chromosome 3 (figure 3*a*, table 1; electronic supplementary material, table S4) when combining the F2 families with EC70 (and for EC70 only; electronic supplementary material, figure S4). The marker explains 15.3% of phenotypic variation in EC70 (which should be an underestimate due to all maternal alleles being ignored) and 9.2% in the three F2 families. Luminance was also strongly associated with markers on chromosome 3 (figure 3*b*; electronic supplementary material, figure S4). The associated marker was 2.75 cM from the marker for BR colour, and the confidence intervals overlap. By contrast, for ridge spacing, we found a significant QTL on chromosome 7 (using just the EC70 brood), explaining 30.3% of variation (figure 3*g*). Again, no significant QTL were found for cross-rib spacing (figure 3*d*). These results were generally supported by a genome-wide association study (GWAS) using all SNP variation (which allowed maternal variation in EC70 to be included) and did not reveal any additional loci (electronic supplementary material, figure S5, see electronic supplementary material for full results and methods).

Individuals with homozygous Panama-type genotypes at the mapped chromosome 3 markers had lower BR and luminance values (figure 3). Individuals carrying Ecuador-type alleles at the mapped chromosome 7 marker showed reduced ridge spacing, consistent with the observation that the Panama subspecies have greater ridge spacing.

### (c) Differential expression

A total of 24 118 genes were expressed in the wings of *H. erato* and 30 721 in the wings of *H. melpomene*. In both *H. erato* and *H. melpomene,* MDS analysis of expression levels revealed strong clustering by stage (dimension 1) and subspecies (dimension 2), leading to four distinct clusters (electronic supplementary material, figure S6). Nine hundred and seven and 1043 genes were differentially expressed (DE) (false discovery rate (FDR) < 0.05) between *H. erato* subspecies at 50% and 70% development, respectively (electronic supplementary material, tables S5 and S6). In *H. melpomene*, 203 and 29 genes were DE between subspecies at 50% and 70% development, respectively (electronic supplementary material, tables S7 and S8). Much of this DE will be due to the genome-wide divergence between subspecies (which is greater in *H. erato* than in *H. melpomene*, [26]), we therefore used further comparisons to narrow down these lists of genes.

Comparing between wing regions, in iridescent *H. erato cyrbia*, there was one gene at 50% and 70 genes at 70% DE (electronic supplementary material, tables S9 and S10); in iridescent *H. melpomene cythera*, there were six genes at 50% and 50 genes at 70% development DE (electronic supplementary material, tables S11 and S12). We may expect that genes involved in scale structure regulation would be DE both between subspecies and wing regions that differ in scale structure, but very few genes were found in both sets (electronic supplementary material, figure S7 and table S13). At 70%, there were two genes upregulated in *H. erato* in both comparisons: *chitin deacetylase 1* has a likely function in the deacetylation of chitin to chitosan and potential structural roles in the cuticle [33], and the other gene has similarity to the circadian clock-controlled gene *daywake*. There was no overlap in significant, downregulated genes expressed at 70% in *H. erato*. At 50% in *H. erato,* there were no significant, concordantly DE genes. However, a *doublesex-*like gene on chromosome 8 narrowly missed the significance cut-off and was downregulated (Log fold change (FC) < −1.5) in both comparisons (FDR = 0.02 between subspecies, FDR = 0.08 between wing regions). In *H. melpomene,* at both 70% and 50%, there was no overlap between genes that were DE between subspecies and wing regions.

Genes involved in controlling scale structure may be similarly DE between species. Between subspecies, at 70%, there were no concordantly DE genes in either species. However, at 50%, there were two concordant genes significantly DE, *Fatty acid synthase* and *Gamma-glutamylcyclotransferase* (electronic supplementary material, table S14). For the wing region comparison, at 70%, there were four concordant genes significantly DE in both species: the homeobox gene *invected, Transglutaminase, uncharacterized LOC113401078* and the *doublesex*-like gene, which was also DE between *H. erato* subspecies (at 50%)*,* but none at 50% (although the *doublesex*-like gene is again DE in *H. melpomene*; electronic supplementary material, table S14).

### (d) Differentially expressed genes in the quantitative trait locus intervals

In order to identify candidate genes in the QTL intervals, we identified DE genes within these genomic regions. In general, the QTL intervals were not significantly enriched for DE genes (based on expected numbers of DE genes for a given interval size; electronic supplementary material, table S15), suggesting that the QTL do not contain clusters of multiple functionally important genes. In *H. erato*, there were two and five DE genes in the ‘BR’ interval on chromosome 20 at 50% and 70% development, respectively (electronic supplementary material, table S15). One of the genes at 70% was *Fringe*, a boundary-specific signalling molecule that modulates the Notch signalling pathway and has roles in eyespot formation and scale cell spacing in butterflies [33,34].

**Figure 4.**
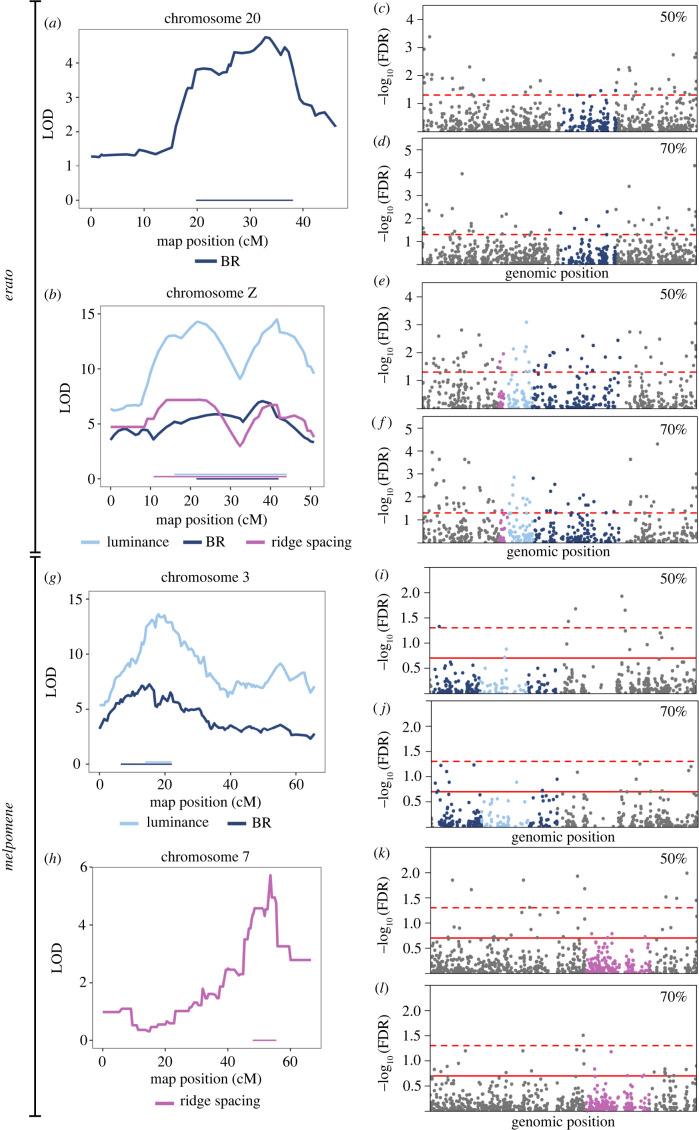
Differential expression of genes in the QTL in *Heliconius erato* (*a–f*) and *Heliconius melpomene* (*g–l*). LOD scores and QTL intervals in *H. erato* (*a,b*) and *H. melpomene* (*g,h*). log_10_FDR for differential expression of all genes on the QTL-containing chromosomes for *H. erato* (*c*–*f*) and *H. melpomene* (*i*–*l*), with genes in the QTL coloured, matching those of the intervals in the panels on the left. In (*e*) and (*f*), the QTL overlap, such that all genes in the BR and ridge spacing intervals also fall within the luminance interval; see electronic supplementary material, table S16 for details. In (*i*) and (*j*) the luminance interval is within the BR interval. The dashed red lines indicate FDR = 0.05 (significance), solid red lines indicate FDR = 0.2.

On the Z chromosome, at 50%, there were 27, 25 and 17 genes significantly DE between subspecies in the ‘ridge spacing’, ‘luminance’ and ‘BR’ intervals, respectively, with 16 genes in the overlap of all three intervals (figure 4; electronic supplementary material, table S16). Of note, the microtubule motor protein, *dynein heavy chain 6* was within all three QTL intervals and highly upregulated (LogFC > 3.0, FDR < 0.05) in the iridescent subspecies. Additionally, an *O-GlcNAc transferase*, with strong similarity to *Drosophila* polycomb group gene *super sex combs* was highly DE (LogFC = −9.32, FDR < 0.004) and matched the exact physical location of the ‘BR’ and ‘luminance’ markers within the genome.

At 70%, on the Z chromosome, there were 24, 23 and 14 genes significantly DE in the ‘ridge spacing’, ‘luminance’ and ‘BR’ intervals, respectively, with 14 shared across all three regions (electronic supplementary material, table S16). The gene *trio*, which functions in actin structure regulation through activation of Rho-family GTPases [33], was found in all three intervals with particular proximity to the ‘ridge spacing’ marker (405 kbp away from the start of this gene). In addition to the functional role of *trio*, its high expression and large fold change (logCPM = 7.34, LogFC = −2.29, FDR = 0.0015) makes it a particularly good candidate for a role in optical nanostructure development in *H. erato*. Furthermore, a novel gene (*MSTRG.21985*) was also DE expressed (LogFC = −1.28, FDR = 0.0115) and may be part of a Rho GTPase activating protein (182 bp upstream of a gene with this annotation).

In *H. melpomene,* there were no DE genes between subspecies in the ‘ridge spacing’ interval on chromosome 7 at either stage. However, at 70%, the gene *ringmaker,* which functions in microtubule organization [33], showed slight DE (logFC = −1.43, FDR = 0.144). On chromosome 3, in the BR interval, there was one novel gene (*MSTRG.3173*) DE at 50% (but this falls outside the luminance interval) and no DE genes at 70% (electronic supplementary material, table S17). The gene *miniature,* which in fly bristles is a component of the cuticulin envelope functioning in interactions between the depositing cuticle, membrane and cytoskeleton [35], falls in the overlap of the luminance and BR regions and shows slight DE at 50% (logFC = 1.60, FDR = 0.192).

For the wing region comparison, in *H. erato*, there were no genes DE at either stage within any of the QTL intervals. For *H. melpomene,* there was one DE gene in the ‘BR’ interval (but outside the ‘luminance’ interval) on chromosome 3 at 70% (a *lactase-phlorizin hydrolase-like* gene) and no DE genes at 50%. For the ‘ridge spacing’ interval on chromosome 7, there was one DE gene at 50%, an *F-actin-uncapping protein LRRC16A* and one gene at 70%, a *cuticle protein 18.6-like* gene (electronic supplementary material, table S18).

## 4. Discussion

In one of the first studies to look at the genetics of structural colour variation in terms of both colour and structure, we show that the trait is controlled by multiple genes in the co-mimics *Heliconius erato* and *Heliconius melpomene*. While we found only a small number of QTL, these explain relatively little of the overall phenotypic variation, suggesting there are more loci that remain undetected. Some of these may be the genes that we detected as DE, but that fall outside the detected QTL intervals. Of particular interest are genes that we detected as DE both between subspecies and between wing regions that differ in scale type. *Chitin deacetylase 1* is one such candidate in *H. erato*, which is on chromosome 5 (not in a QTL interval). Chitin is the main component of the cuticle and the differential expression of a potential chitin-degrading gene could alter the formation of the scale ridges [36].

Within each species, we find that hue and brightness (BR and luminance) are controlled by loci on the same chromosomes. In *H. erato*, this was on the Z chromosome, confirming our previous phenotypic analysis [23], and in *H. melpomene*, on chromosome 3. An additional locus on chromosome 20 was also found to affect blue colour but not brightness in *H. erato*. The Z chromosome locus in *H. erato* appears to control ridge spacing, which could have a direct effect on the brightness of the reflectance by increasing the density of reflective structures. Indeed, in the single-family analyses, luminance and ridge spacing mapped to exactly the same marker. However, the observed correlation between brightness and ridge spacing in *H. erato* may be a product of an unobserved association between tighter ridge spacing and other aspects of scale nanostructure, specifically the number of lamellae layers within the ridges. Theoretical analyses and simulations of the optical properties of multi-layers have revealed that increasing the number of layers will result in a rapid increase of brightness; adding even a small number of layers produces a significant increase in the amount of reflected light [37]. Therefore, the Z chromosome locus may be affecting multiple aspects of scale structure, producing the observed correlations between the different colour and structure measurements. Indeed, some DE genes in the Z locus may control multiple aspects of scale structure. For example, *trio* acts in several signalling pathways to promote reorganization of the actin cytoskeleton through Rho GTPase activation. Its regulatory function may be repeatedly employed during scale development in the formation of different aspects of scale ultrastructure guided by the actin cytoskeleton. Interestingly, potentially related signalling genes, such as the novel gene located immediately before a Rho GTPase activating protein, also fall within this locus and are DE, potentially suggesting there are several functional genes linked together in this region.

By contrast, in *H. melpomene*, we found different loci controlling colour and ridge spacing, suggesting a more dispersed genetic architecture and different loci controlling different aspects of scale structure. We found strong evidence for a locus on chromosome 3 controlling BR and luminance, but this locus appeared to have no effect on our measurements of scale structure and so is likely controlling other aspects of scale structure not quantified here. Instead, we find a locus on chromosome 7 that partially controls ridge spacing. Combined with the lack of a correlation between ridge spacing and our colour measurements, it appears that ridge spacing has relatively little direct effect on colour in *H. melpomene*, despite the parental populations showing a similar difference in ridge spacing to that seen in *H. erato*. It appears that *H. erato* has a locus on the Z chromosome that can control multiple aspects of scale structure, while scale structure variations in *H. melpomene* involve mutations at loci dispersed around the genome. This could provide one explanation for how *H. erato* has been able to evolve brighter structural colour than that observed in *H. melpomene*, if single mutations in *H. erato* can have pleiotropic effects on multiple aspects of scale structure.

In contrast with many of the loci for pigment colour patterns that are homologous across multiple *Heliconius* species, the loci controlling iridescence in *H. erato* and *H. melpomene* appear to be largely different. Differences in the physical scale architecture and brightness of colour between the species perhaps make these genetic differences unsurprising [16,26]. A lack of genetic parallelism may also be more likely for a quantitative trait such as iridescence [29]. Nevertheless, on the Z chromosome in *H. melpomene*, we do observe elevated LOD scores in the QTL analysis and low *p*-value SNPs in the GWAS for both scale structure traits, but neither of the colour traits. This suggests that *H. melpomene* may have a locus homologous to that in *H. erato,* which is controlling some aspects of scale structure variation, but with apparently little or no effect on colour variation. In addition, we find some genes that appear to show parallel expression patterns between species. Of particular interest is a *doublesex*-like gene that is DE between wing regions in both species and between *H. erato* subspecies. A different duplication of *doublesex* has been found to control structural colour in the Dogface butterfly (*Zerene cesonia*) [38], making this an interesting, potentially parallel candidate between species. It is possible that the evolutionary pathways may be different between species, but have triggered expression changes in similar downstream developmental pathways. However, we found very few genes that show concordant expression patterns between species.

In recent years, reverse genetics research has revealed a surprising connection between the molecular machinery underlying the development of pigmented wing patterns and the ultrastructure of butterfly scales in various species [17,18,39,40]. However, our QTL are not associated with any known colour pattern gene of large or small effect in *Heliconius* (*aristaless, WntA, vvl*, *cortex* and *optix—*located on chromosomes 1, 10, 13, 15 and 18, respectively) [22]. Our findings show that *H. erato* and *H. melpomene* do not use the known molecular machinery of wing pattern production for sculpting specialized nanostructures and iridescent wings, and that the production of structural colour is completely decoupled from that of mimicry-related wing pattern regulation and pigment production.

Overall, we show major differences in the genetic basis of structural colour in *H. erato* and *H. melpomene*. Combining this with gene expression analyses, we have been able to identify novel candidate genes for the control of structural colour variation with potential functions in chitin metabolism, cytoskeleton formation, gene expression regulation and cell signalling.

## Data Availability

Genomic sequence data from the crosses are deposited in the European Nucleotide Archive under project no. PRJEB38330. RNA sequence data are deposited in the Gene Expression Omnibus (GEO) with series accession GSE190378. Photographs of all samples can be found at doi.org/10.5281/zenodo.3799188. USAXS data are available at doi.org/10.5281/zenodo.5747416 and handling scripts at https://github.com/juanenciso14/butterfly_usaxs. Linkage maps with all phenotypic measurements and QTL scripts are available at github.com/mnbrien/Heliconius-iridescence. The data and an abstract in Spanish are provided in the electronic supplementary material [41].
